# Axillary Metastasis as the Initial Presentation of Recurrent Nasopharyngeal Carcinoma

**DOI:** 10.7759/cureus.58625

**Published:** 2024-04-20

**Authors:** Muhammad Adzha Musa, Ahmad Nordin Afandi, Farah Dayana Zahedi

**Affiliations:** 1 Department of Otorhinolaryngology-Head and Neck Surgery, Hospital Canselor Tuanku Muhriz, Kuala Lumpur, MYS; 2 Department of Otorhinolaryngology, Hospital Queen Elizabeth, Kota Kinabalu, MYS

**Keywords:** lymph node metastasis, axillary swelling, axillary lymph nodes, recurrent nasopharyngeal carcinoma, nasopharyngeal cancer (npc)

## Abstract

The recurrence of nasopharyngeal carcinoma (NPC) is either at the local primary site or at regional or distant metastases. However, an axillary metastasis is a rare entity in NPC. We highlighted a case of recurrent NPC that presented with axillary swelling as the main initial complaint. Clinical examinations showed enlarged left axillary lymph nodes and left cervical lymph nodes. Histopathological examination of the axillary lymph node biopsy confirmed the recurrence of NPC. The patient underwent palliative chemotherapy in view of the advanced stage of recurrent disease. A thorough clinical history and examination during surveillance are crucial for early diagnosis and better survival outcomes.

## Introduction

Nasopharyngeal carcinoma (NPC) is a malignant epithelial tumor of the nasopharynx. It has the highest prevalence in the regions of Southeast Asia, southern China, and North Africa and represents 5.2% of all cancers in Malaysia [1]. Nasopharyngeal carcinoma is a radiosensitive tumor, and radiotherapy has been the main treatment modality in the newly diagnosed NPC. However, NPC can recur, and distant metastasis is commonly due to failure of treatment in NPC, with an overall five-year survival rate of 46% [1]. Nasopharyngeal carcinoma with axillary lymph node metastases has rarely been reported in the literature. To date, there is only one case of recurrent NPC with axillary metastasis reported in the literature. We hereby highlight a case of recurrence of NPC with swelling of the axillary lymph nodes as an initial presentation. 

## Case presentation

A 55-year-old gentleman with underlying NPC presented with painless swelling of the left axillary for two months. He was previously diagnosed with stage III NPC (T1N2M0) three years ago, in which he presented swelling in the left neck for two months. A biopsy of the left fossa of the Rosenmuller mass confirmed the diagnosis of undifferentiated, non-keratinizing squamous cell carcinoma of the nasopharynx. Subsequently, he defaulted on treatment for a year due to logistical and financial issues and returned with a growing swelling in the left neck a year later. Re-staging of the disease was performed and confirmed the progression of the disease to stage IVA (T2N3M0). He completed concurrent chemo-radiotherapy and was on regular monthly follow-up with no signs of residual or recurrent disease. After nine months of completion of the treatment, he complained of painless enlargement of the left axillary for two months, followed by enlarging left neck swelling one month later. He denied any nasal or ear symptoms. 

**Figure 1:**
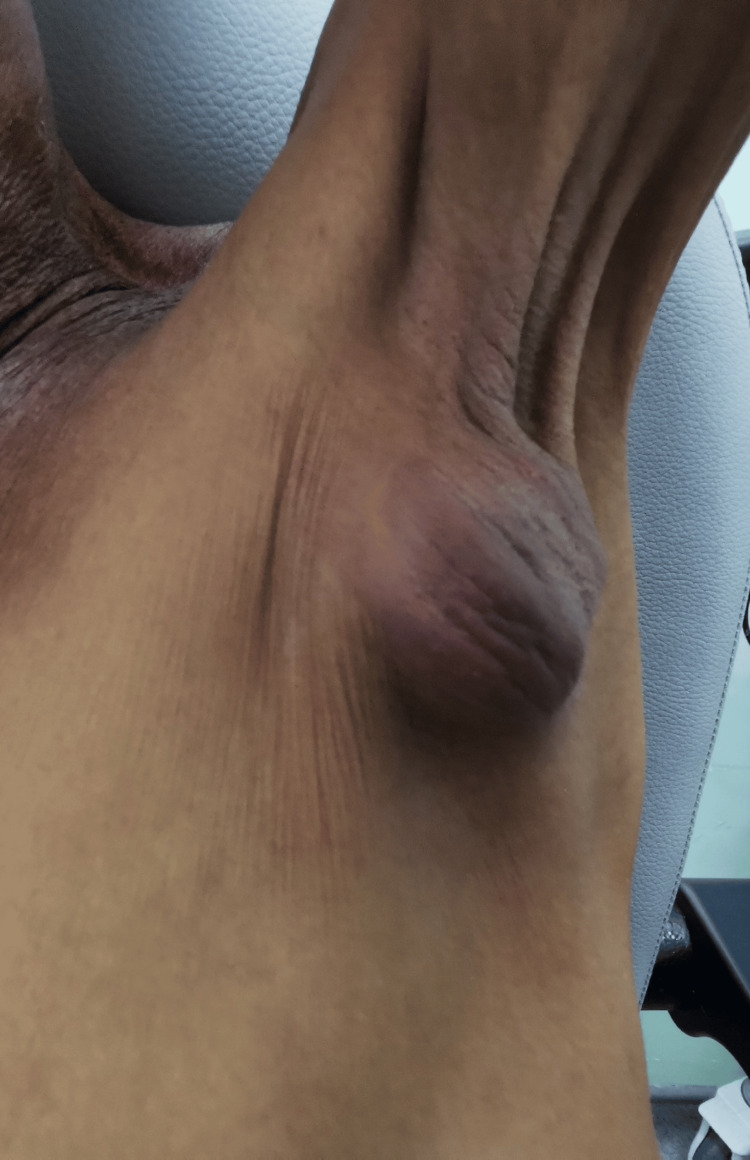
Clinical image showing a 4x4 cm left axillary swelling

On examination, there was an axillary swelling measuring 4x4 centimeters (cm), firm, mobile, and non-tender (Figure 1). There was also a palpable, fixed hard left cervical lymph node at levels II and III measuring 3x3 cm. The nasal endoscopic examination did not show evidence of local recurrence. Fine needle aspiration and cytology (FNAC) of the left cervical lymph nodes and the swelling of the left axillary region revealed atypical cells displaying oval nuclei with mild nuclear pleomorphism, and no evidence of malignant cells was seen. In view of the inconclusive result of FNAC, core needle biopsies of the left axillary swelling and left cervical lymph node were performed, and histopathological examination of the left axillary swelling showed malignant cells that exhibited moderate nuclear pleomorphism and large nucleoli, arranged in syncytial sheets, nests, and trabeculae patterns that suggested metastatic NPC.

**Figure 2:**
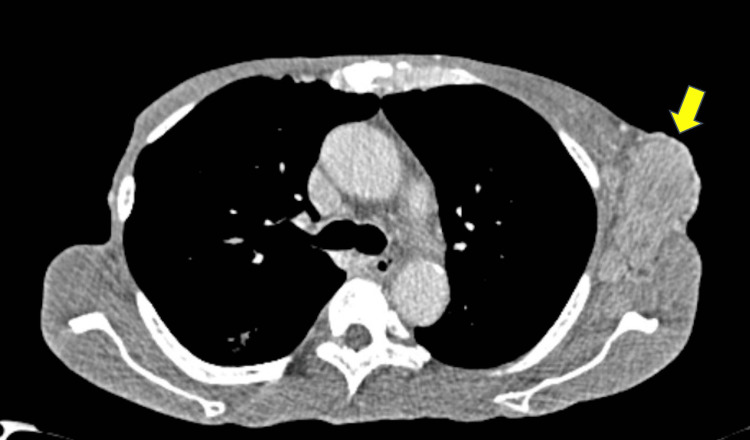
Computed tomography image shows an enlarged left axillary lymph node measuring 6x3 cm (yellow arrowhead)

A contrasted computed tomography (CT) scan of the neck, thorax, and abdomen showed obliteration of the bilateral fossa of Rosenmuller, multiple enlarged left cervical lymph nodes and mediastinal nodes, and bilateral axillary nodes with distant metastases to the lung, liver, and spleen (Figure 2). He was diagnosed with a recurrence of NPC with distant metastases and was referred to the oncology team for palliative chemotherapy. Unfortunately, he did not respond to palliative chemotherapy and passed away due to the progression of the disease. 

## Discussion

The recurrence of NPC can be local, regional, or distant metastases. It has been well documented that those with advanced stages at the time of initial diagnosis have a poorer prognosis and a higher risk of recurrence. Identifying prognostic factors for treatment failure in NPC is important in predicting the risk of recurrence. It has been well established that male gender and tumors with nodal involvement have also been shown to have a poorer prognosis [2]. It has also been well established that a high pretreatment plasma level of Epstein-Barr virus (EBV)-DNA of more than 4,000 copies/mL and overall tumor, node, metastasis (TNM) staging, especially high staging of N, are associated with a poor prognosis. Recent studies have also shown that a high absolute monocyte count and an absolute lymphocyte count during treatment were associated with a poor prognosis in patients with NPC [2]. Our patient is an elderly gentleman who had high N staining during initial staging. There was no pretreatment plasma EBV-DNA level taken because the test was not routinely performed due to its unavailability in our center. 

The most common pattern of NPC recurrence was local (73.5%), followed by locoregional lymph nodes (21.7%) and distant metastasis [3]. Bone, lung, and liver are common sites of distant metastases, and supraclavicular nodes are the most advanced extent of locoregional spread reported [4]. A recent retrospective study involving 2,599 NPC patients found that 59% of recurrent NPC occurs within the first two years after completion of treatment [5]. Axillary lymph node metastasis in NPC is uncommon and rarely reported in the literature. A retrospective study conducted at the Memorial Sloan-Kettering Cancer Center in 2013 reported that only 8% of cases with axillary metastases were seen in head and neck carcinoma, but none of the cases were NPC [6].

There are many theories that explain the mechanism of axillary metastasis in NPC. Axillary lymph node metastasis in NPC is best explained by the cephalad-caudal pattern of nodal spread, suggesting that the axillary nodes and superior mediastinal nodes are the next possible tumor spread stations after the supraclavicular nodes [3]. The other theory is closely related to the previous radiation therapy. It has been proposed that previous radiation therapy has decreased the local blood supply adjacent to the nasopharynx, which is unfavorable for tumor growth, and caused the tumor to relocate at a site far from the nasopharynx, such as the axilla [7]. 

18F-fluorodeoxyglucose positron emission tomography (FDG-PET) is the best diagnostic modality for the recurrence of NPC, with a combined sensitivity of 95% and a specificity of 90% [1]. However, a detailed clinical history and examinations still play a pivotal role in suspecting a recurrence of NPC. A deep-seated or submucosal NPC may not be detected on endoscopy, and CT or magnetic resonance imaging (MRI) may be useful in this situation. Magnetic resonance imaging is much better than CT in detecting intracranial and skull base infiltration and differentiating recurrent disease from post-radiation tissue changes [1, 8]. However, the image-based diagnosis of recurrent NPC may still be unreliable due to false positivity, and the findings should be correlated with clinical findings when suspecting recurrent NPC. In our case, no FDG-PET was performed due to the inaccessibility of the FDG-PET scan at our center, and the diagnosis was confirmed by histopathological examination of the core needle biopsy of left axillary swelling. 

The treatment modalities for recurrent disease are determined by local, locoregional, or distant recurrence. Treatment options for local recurrence can be either nasopharyngectomy, reirradiation, or brachytherapy, depending on the T-staging of the disease. The overall five-year survival rate of endoscopic nasopharyngectomy in recurrent T1 and T2 diseases was reported to be 84.6% [9]. Re-irradiation therapy for recurrent NPC has proven to be challenging due to the fact that the effective radiation dose that can be administered is limited due to previous radiation and long-term toxicity [10]. Intensity-modulated radiation therapy has been recommended as the preferred modality for reirradiation in recurrent NPC [11]. The treatment options for locoregional nodal recurrence are neck dissection, reirradiation, and chemotherapy, while in recurrent NPC with distant metastases, treatment options include palliative chemoradiation or palliative care. Currently, there is a paucity of evidence in the literature on the outcome of axillary dissection in recurrent nasopharyngeal carcinoma, and therefore chemoradiation and palliative care remain the options of treatment in recurrent nasopharyngeal carcinoma with axillary metastasis. 

## Conclusions

Axillary lymph node metastasis in recurrent NPC is a rare occurrence, and it is uncommon to have axillary swelling as an initial presentation of recurrent NPC. A thorough clinical history and examinations with the aid of diagnostic imaging tools and tissue biopsy are essential in diagnosing recurrent NPC. The first two years after the completion of treatment are the critical period of surveillance, and early detection is important to improve survival outcomes. 
